# Language, cognitive, and speech in noise perception abilities of children with cochlear ımplants: a comparative analysis by implantation period and bilateral versus unilateral cochlear implants

**DOI:** 10.1007/s00405-024-08462-x

**Published:** 2024-01-20

**Authors:** Merve İkiz Bozsoy, Esra Yücel

**Affiliations:** Department of Audiology, Faculty of Health Sciences, Hacettepe University, 06100 Ankara, Turkey

**Keywords:** Early cochlear implant, Bilateral cochlear implant, Language skills, Cognitive skills, Speech in noise perception

## Abstract

**Purpose:**

The purpose of this study was to compare the language, cognitive, and speech in noise (SiN) perception abilities of children with cochlear implants (CIs) to those of their peers with NH by grouping them according to their implantation period (12–18 months/19–24 months) and unilateral/bilateral CI use.

**Methods:**

The sample comprised 50 children with cochlear implants (CIs) and 20 children with normal hearing (NH), ages 6–9 years. Children’s language, cognitive, and speech in noise (SiN) perception skills were assessed.

**Results:**

Children with CIs between 12 and 18 months and 19 and 24 months performed more poorly than children with NH on language, verbal memory (VM), verbal-short-term memory (V-STM), verbal working memory (V-WM), rapid naming, and speech in noise (SiN) perception abilities measures (*p* < 0.001). In addition, children with CIs between 19 and 24 months performed worse on rapid naming and V-WM tasks than children with CIs between 12 and 18 months (*p* < 0.017). Children with unilateral and bilateral CI performed more poorly than children with NH on language, VM, V-STM, V-WM, rapid naming, and SiN perception abilities assessments (*p* < 0.001). Additionally children with unilateral CI users performed poorly than children with bilateral CI users on SiN perception (*p* < 0.017).

**Conclusions:**

In children with congenital hearing loss (CHL), cochlear implantation between 12 and 18 months or sequential bilateral implantation is not sufficient for these children to perform like their NH peers in language, cognitive, and SiN perception abilities. In addition, intervention approaches should focus not only on increasing language skills, but also on cognitive abilities.

## Introduction

Auditory experiences play critical role in individual’s language and neurocognitive functioning [1]. Several studies have shown that severe to profound hearing loss (SPHL) affects speech and language development negatively [2, 3]. In addition, it is well established that prelingual HL affects almost all aspects of general development, including cognitive, motor, and social domains [4].

To prevent these negative effects, CIs are optimized for pediatric users. The CIs significantly improves language abilities, cognitive skills like memory, organization, discrimination, and SiN perception abilities [4]. However, there have been some important factors shaping post-CI performance, such as age at diagnosis, additional disability, age of implantation, bilateral CI use, and parental involvement [5–8].

A lack of early auditory experience alters the normal processes of synaptogenesis and pruning, hence impairing functional maturation. Early intervention during sensitive period is the only way to prevent further deterioration in the functional maturation of the central nervous system [1]. Children implanted in the early period represent the groups who have access to auditory information during the sensitive developmental period [9]. Early access to auditory cues facilitates not only language development but also other domains of development. Because, during the first 3–4 years of life is the time of the highest central auditory nervous system (CANS) plasticity and prelingual HL affects the whole central nervous system [4]. Therefore, existing research suggests that the earlier implantation, the better the language, cognitive, and SiN perception skills [10–12].

The use of bilateral CIs is another significant factor influencing outcomes. The majority of noise reduction and acoustical orientation abilities of the human auditory system depend on the listener’s access to time, level, and spectral differences between sound signals perceived by the two ears. Thus, unilateral CI for bilaterally deaf children improve language development but not binaural development [13]. Therefore, early and bilateral access to hearing is essential for the maturation of the brain's auditory system, and a lack of auditory stimulation can cause neural development deviations that affect auditory development, language acquisition, and even higher level cognitive abilities [7].

In our country, the earliest age for implantation is 12 months, as the state covers the cost of the CI systems for children 12 months and older with bilateral SPHL and limited benefit of binaural hearing aids (HAs) [14]. Additionally, bilateral CIs have been applied simultaneously or sequentially to children aged 12–48 months in our country, since 2016 [15]. In the literature, there is a lack of evidence regarding when early cochlear implantation will be more effective in improving children’s performance. Several studies have shown that implanting children until to 18 months of age provides better speech recognition outcomes than later implantations [16, 17]. A large number of studies also indicate a significant decrease in hearing and speech outcomes if children are implanted after the age of 24–36 months [18, 19]. Additionally, while multiple studies have described the clear advantages of bilateral implantation over unilateral implantation in terms of SiN perception [20–22], a few studies have studied how they perform in comparison to NH peers [23, 24]. There is also a small number of studies comparing the cognitive and linguistic abilities of children with unilateral and bilateral CIs [25, 26]

In line with these informations, this study has two aims: (1) To compare the language, cognitive, and SiN perception abilities of children with CIs between 12 and 18 months, with CIs between 19 and 24 months, and with normal hearing (NH). (2) To compare the language, cognitive, and SiN perception abilities of children with unilateral CI (UnCI) users, with bilateral CIs (BiCIs) users, and with NH.

## Materials and methods

This study was conducted at Hacettepe University Faculty of Health Sciences, Audiology Department and received ethical approval from the Hacettepe University Clinic Research Ethics Board (KA-21051). The participants and their parents provided informed consent on the day of enrollment.

### Participants

Fifty children with CIs and 20 children with NH were included in the study and control group, respectively. The following inclusion criteria were used to determine the study group: (1) receiving their CIs in Hacettepe University Hospital, (2) using UnCI or BiCIs, (3) failing in the newborn hearing screening, (4) having prelingual SPHL, (5) participants with UnCIs; receiving their CI within 24 months, and not wearing a contralateral HA, (6) participants with BiCIs; undergoing the first CI surgery for up to 24 months, receiving their CIs sequentially, and utilizing BiCIs for at least 12 months, (7) receiving regular implant mapping at our center at least annually, (8) receiving regular special education, and (9) sound field hearing thresholds with UnCI or BiCIs within the frequency-intensity field of speech. Apart from these, children diagnosed with inner ear malformations, auditory neuropathy spectrum disorders, neurological or developmental disorders, learning difficulties, and other comorbidities were excluded. Furthermore, since BiCIs surgery started in our country in 2016, the number of children who received simultaneous BiCIs and met the inclusion criteria was highly limited at the time of this study. Accordingly, simultaneous BiCIs users could not be included in this study.

The control group included children who met the following criteria: (1) passing the newborn hearing screening (2) have hearing thresholds ≤ 20 at octave frequencies 0.25–8.0 kHz, (2) no developmental or neurocognitive delays or disabilities.

When the study group was divided by first implantation period (12–18 months vs. 19–24 months), the number of participants who received a CI between 12 and 18 months (12–18 CI) and 19 and 24 months (19–24 CI) was 24 (12 F, 12 M) and 26 (14 F, 12 M), retrospectively. Comparative statistical analysis showed that there were no statistically significant differences in gender and chronological age between the 12 and 18 CI, 19 and 24 CI, and NH groups. However, a significant difference was found in terms of paternal and maternal education. In the comparison between the groups with 12–18 CI and 19–24 CI, while there were no significant differences in terms of the number of UnCI/BiCIs users and age at diagnosis, significant differences were found in the age of implantation and hearing aided age.

When the study group was divided by UnCI/BiCIs, the number of UnCIs and BiCIs users was 23 (11F, 12 M) and 27 (15 F, 12 M), respectively. Comparative statistical analysis showed that there were no statistically significant differences in gender, chronological age, paternal education level, and maternal education level between the groups. Also, there were no significant differences between the UnCIs and BiCIs users in terms of the age at diagnosis, age of implantation, and hearing aided age.

Table 1 provides descriptive and comparative statistics of the demographics and hearing history of the groups.

**Table 1:** Demographics and hearing history of the participants

Total study population (*N* = 70)								
	12–18 CI (*n* = 24)	19–24 CI (*n* = 26)	NH (*n* = 20)		UnCIs (*n* = 23)	BiCIs (*n* = 27)	NH (*n* = 20)	
	*n* (%)	*n* (%)	*n* (%)	*p*	*n* (%)	*n* (%)	*n* (%)	*p*
Gender				0.95				0.85
Female	12 (50)	14 (53)	10 (50)		11 (47.8)	15 (55.6)	10 (50)	
Male	12 (50)	12 (47)	10 (50)		12 (52.2)	12 (44.4)	10 (50)	
Unilateral/bilateral				0.36	–	–	–	–
Unilateral	13 (54.2)	10 (38.5)	–		–	–	–	–
Bilateral	11 (45.8)	16 (61.5)	–		–	–	–	–
Maternal education				**0.025**				0.20
Primary school	9 (37.5)	19 (73.1)	6 (30)		14 (60.9)	14 (51.9)	6 (30)	
High school	11 (45.8)	6 (23.1)	12 (60)		8 (34.8)	9 (33.3)	12 (60)	
Bachelor degree	4 (16.7)	1 (3.8)	2 (10)		1 (4.3)	4 (14.8)	2 (10)	
Paternal education				**0.029**				0.06
Primary school	6 (25)	11 (42.3)	1 (5)		9 (39.1)	8 (29.6)	1 (5)	
High school	9 (37.5)	9 (34.6)	14 (70)		8 (34.8)	10 (37)	14 (70)	
Bachelor degree	9 (37.5)	6 (23.1)	5 (25)		6 (26.1)	9 (33.3)	5 (25)	

	*M* (SD)	*M* (SD)	*M* (SD)	*p*	*M* (SD)	*M* (SD)	*M* (SD)	*p*
Age (y)	7.37(0.82)	7.06 (1.02)	7.43(0.97)	0.25	7.31 (0.91)	7.12 (0.96)	7.43 (0.97)	0.49
Age of diagnosis (m)	5.04 (2.96)	6.19 (4.30)	–	0.42	5.78 (3.07)	5.52 (4.26)	–	0.38
Implantation age (y)	1.23 (0.16)	1.81 (0.14)		**0.00**	1.49 (0.27)	1.57 (0.37)	–	0.33
Hearing aided age (y)	7.58 (3.35)	10.46 (4.48)	–	**0.014**	9.22 (3.38)	8.96 (4.85)	–	0.83
Age at special education (m)	8.25 (2.19)	11.23 (2.91)	–	** < 0.001**	9.52 (2.87)	10.03 (3.09)	–	0.65

Bold indicates significant difference

### Test battery

#### Audiological assesment

Pure-tone audiometry was first performed within inclusion criteria after informed consent and a detailed history. In children with NH, air- and bone-conduction thresholds were measured at octave frequencies between 0.25 and 8 kHz using TDH-39 supra-aural earphones and bone vibrator. In children with CIs, pure-tone thresholds with the implant were measured at octave frequencies between 0.25 and 8 kHz in the sound field. Each implant's threshold was measured for children with bilateral CIs.

#### Language measurement

Participants' language skills were measured by Test of Language Development-primary: Fourth edition (TOLD-P:4). It was developed by Phyllis L. Newcomer and Donald D. Hammill in the United States [27]. The normative data for the Turkish version of the TOLD-P:4 consisted of 1252 individuals aged 4–8 years 11 months. The validity and reliability results are strong and significant. The test's accuracy in identifying typically developing and language-impaired children was also tested. The positive and negative likelihood ratios, as well as the sensitivity and specificity analyses, were computed. For subtests of the TOLD-P:4 Turkish version, the data-based cut-off point was determined to be – 1.00 SD or 85 quotient. The results showed that the Turkish version of the TOLD-P:4 accurately identified children with typical language development and those with language deficits.

The TOLD-P:4 subtests enable evaluation of the linguistic components. It consists of six core subtests (picture vocabulary-PC, relational vocabulary-RV, word description-WD, sentence comprehension-SC, sentence repetition-SR, and morpheme completion-MC) and three complementary tests (word differentiation, phonological analysis, and word production). Some core subtests scores are combined, and listening, organizing, speaking, grammar, and semantic indexes scores are obtained. The listening index score consists of PV and SC scores. The organizing index score consists of RV and SR scores. Speaking index score consists of WD and MC scores. The grammar index score consists of SC, SR, and MC scores. The semantic index score consists of PV, RV, and WD scores. Combining six core subtest scores offers an oral language index score. According to the index scores, descriptive categories in the healthy group are determined as very poor, poor, below average, average, and above average [28].

#### Cognitive Measurements

*Working Memory Scale* Working Memory Scale (WMS) was developed by Ergul et al. [29] to evaluate the WM performance of children aged 5–10 years old. The scale comprises nine subtests across four dimensions, including verbal/visual short-term memory and verbal/visual working memory. WMS's validity and reliability sample included 1494 children. Expert opinions assessed scale content validity. Principal content, cluster, and confirmatory factor analyses assessed construct validity. Exploratory and confirmatory analyses arranged the number of items for each subscale to maximize structural validity. Test–retest reliability was between 0.41 and 0.75. Cronbach Alpha coefficients between 0.66 and 0.84 indicated strong internal consistency.

In this study, participants completed the Verbal Memory subscale's V-STM and V-WM subtests. Digit, Word, and Nonword Recall tests evaluate V-STM. Backward Digit and First Word Recall tests evaluate V-WM. During the application of the subscales, the sequences in each of the items are presented sequentially to the child, the child is asked to repeat the stimulus they hear in the same or reverse order. Each test has two trials and escalating sequences. If at least one trial in each item is successful, the child can pass to the next item. If both trials fail, the test terminates. Each test item answered correctly receives 1 point. The child must correctly repeat all numbers and words to receive points. After subtests, V-STM, V-WM, and verbal memory (VM) scores are calculated. The child's V-STM, V-WM, and VM developmental level relative to typically developing peers (very low, low, medium, high, and very high) is determined by the subscale scores.

*The Rapid Naming Test* The Rapid naming test (RNT) measures memory access to phonological information. Ergül et al. [30] developed the RNT, because rapid naming is related to several cognitive skills. It has four subtests: object, color, letter, and digit naming. The construct validity of the four subtests was tested with explanatory factor analysis and confirmatory factor analysis. The analysis showed that each subtest was variable and had discrimination validity. The-continuity-stability reliability was examined by test–retest method and high correlation values were found for all subtests (0.83–0.95).

The RNT subtests had five rows of ten items each. The test items are introduced to the child before each subtest. The five elements are then presented in a random order. When the child names the first object, the stopwatch starts and stops when the child name the last item. The child's subtest score is the time to name all items. According to the typically developing peers, the total naming time is determined as very slow, slow, average, rapid, or very rapid. In this study, we used object naming subtest.

#### Speech in Noise Measurement

This study assessed SiN perception using Turkish Hearing in Noise Test-Children (THINT-C). THINT-C sentences were phonetically balanced from elementary school texts**.** The 120 sentences with the highest accuracy scores were identified and divided into 12 phonemically matched 10-sentence lists [31]. This study will refer to the THINT-C as HINT for brevity.

The participants’ performances were evaluated with a speech and noise stimulus from a 1 m-away speaker at 0 azimuths in anechoic chamber. Participants were asked to repeat three sets of ten sentences selected at random by the software. Soli and Wong's adaptive HINT procedure [32] was used to determine the SNR: The noise level is fixed at 65 dBA and the software adjusted the intensity of the speech stimulus based on participant responses. The first sentence was presented at 0 dB SNR, followed by 4 dB steps for the next 4 sentences and 2 dB steps for the rest. The SRT was calculated as the mean SNR at which the participant could correctly repeat %50 of the sentences. The SRTs of the 3 lists were averaged.

The HINT test was administered to bilateral CIs users in both the condition of their first implant and the condition of bilateral use. The first implant score was included in implantation period comparisons, while the bilateral score was included in unilateral/bilateral CIs use comparisons.

### Statistical analysis

SPSS version 23 was used to analyze data. The variables' normality was determined using histograms, probability plots, and Kolmogorov–Smirnov/Shapiro–Wilk's test. Means, standard deviations, and percentages were used for descriptive analysis. For parametric and non-parametric comparisons, Independent sample *t* test and Mann–Whitney *U* test were used. Kruskal–Wallis tests and Mann–Whitney *U* tests with Bonferroni correction were used to compare three groups. Chi-Squared analysis compared categorical variables. *p* < 0.05 indicated statistical significance.

## Results

### Comparisons by implantation period

**Fig. 1:**
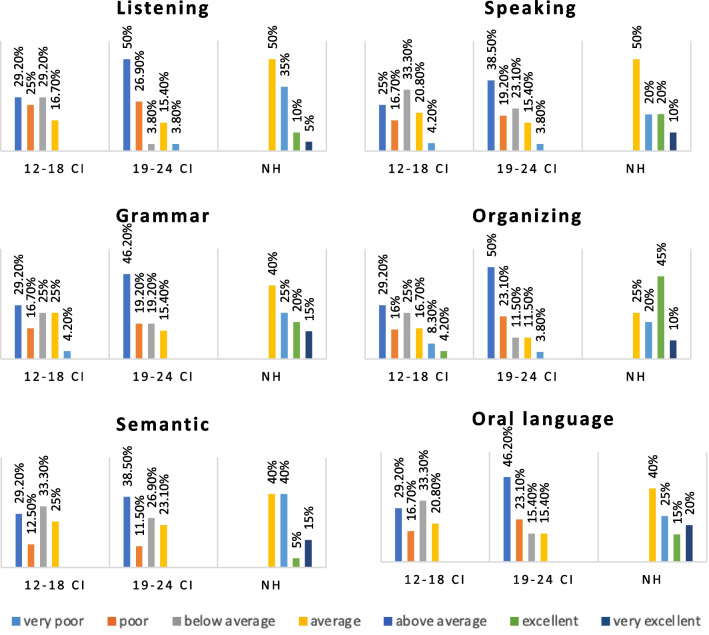
Comparison of TOLD-P:4 category indexes according to implantation period

The 12–18 CI, 19–24 CI, and NH groups revealed significant differences (*p* < 0.001) in the TOLD-P:4 indexes (listening, organizing, speaking, grammar, semantic, and oral language). In the post hoc analysis, the categories of the 12–18 CI and 19–24 CI were not significantly different (*p* > 0.017); however, the categories of the NH were significantly higher (*p* < 0.017) (Fig. 1, Table 2).

**Table 2:** The Kruskal–Wallis analysis of TOLD-P:4 indexes categories and post hoc results

According on implantation period		According on unilateral/bilateral CI use	
	*p*		*p*
Listening	< 0.001*		< 0.001*
12–18 CI × 19–24 CI	0.16	Unilateral × bilateral	0.71
12–18 CI × NH	< 0.001*	Unilateral × NH	< 0.001*
19–24 CI × NH	< 0.001*	Bilateral × NH	< 0.001*
Organizing	< 0.001*		< 0.001*
12–18 CI × 19–24 CI	0.06	Unilateral × bilateral	0.29
12–18 CI × NH	< 0.001*	Unilateral × NH	< 0.001*
19–24 CI × NH	< 0.001*	Bilateral × NH	< 0.001*
Speaking	< 0.001*		< 0.001*
12–18 CI × 19–24 CI	0.29	Unilateral × bilateral	0.08
12–18 CI × NH	< 0.001	Unilateral × NH	< 0.001
19–24 CI × NH	< 0.001*	Bilateral × NH	< 0.001*
Grammar	< 0.001*		< 0.001*
12–18 CI × 19–24 CI	0.12	Unilateral × bilateral	0.25
12–18 CI × NH	< 0.001*	Unilateral × NH	< 0.001*
19–24 CI × NH	< 0.001*	Bilateral × NH	< 0.001*
Semantic	< 0.001*		< 0.001*
12–18 CI × 19–24 CI	0.57	Unilateral × bilateral	0.02
12–18 CI × NH	< 0.001*	Unilateral × NH	< 0.001*
19–24 CI × NH	< 0.001*	Bilateral × NH	< 0.001*
Oral language	< 0.001*		< 0.001*
12–18 CI × 19–24 CI	0.15	Unilateral × bilateral	0.11
12–18 CI × NH	< 0.001*	Unilateral × NH	< 0.001*
19–24 CI × NH	< 0.001*	Bilateral × NH	< 0.001*

Bold inidcates significant difference

**Fig. 2:**
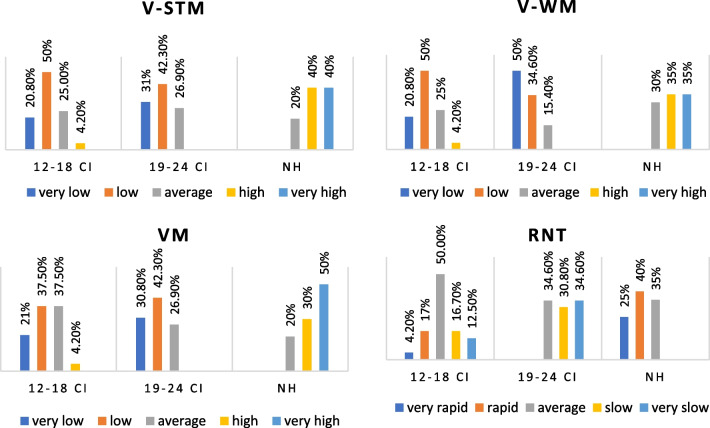
Comparison of VMS and RNT levels according to implantation period

The WMS assessment showed that the groups had different STM, WM, and VM levels. The post hoc analysis showed no significant difference between the STM and VM levels of the 12–18 CI and 19–24 CI groups (*p* > 0.017); however, the NH group's level was significantly greater (*p* < 0.017). On the WM subtest, the 12–18 CI group outperformed the 19–24 CI group (*p* < 0.017), and the NH group outperformed both groups (*p* < 0.017). (Fig. 2, Table 3).

**Table 3:** The Kruskal–Wallis analysis of VMS and RNT levels, and HINT scores of the groups and post hoc results

According on implantation period		According on unilateral/bilateral CI use	
	*p*		*p*
V-STM	< 0.001*		< 0.001*
12–18 CI × 19–24 CI	0.51	Unilateral × bilateral	0.17
12–18 CI × NH	< 0.001*	Unilateral × NH	< 0.001*
19–24 CI × NH	< 0.001*	Bilateral × NH	< 0.001*
V-WM	< 0.001*		< 0.001*
12–18 CI × 19–24 CI	0.004*	Unilateral × bilateral	0.15
12–18 CI × NH	< 0.001*	Unilateral × NH	< 0.001*
19–24 CI × NH	< 0.001*	Bilateral × NH	< 0.001*
VM	< 0.001*		< 0.001*
12–18 CI × 19–24 CI	0.23	Unilateral × bilateral	0.08
12–18 CI × NH	< 0.001*	Unilateral × NH	< 0.001*
19–24 CI × NH	< 0.001*	Bilateral × NH	< 0.001*
RNT	< 0.001*		< 0.001*
12–18 CI × 19–24 CI	0.004*	Unilateral × bilateral	0.25
12–18 CI × NH	< 0.001*	Unilateral × NH	< 0.001*
19–24 CI × NH	< 0.001*	Bilateral × NH	< 0.001*
HINT	< 0.001*		< 0.001*
12–18 CI × 19–24 CI	0.097	Unilateral × bilateral	0.002*
12–18 CI × NH	< 0.001*	Unilateral × NH	< 0.001*
19–24 CI × NH	< 0.001*	Bilateral × NH	< 0.001*

Bold inidcates significant difference

The RNT results showed a statistically significant difference in rapid naming between groups (*p* < 0.05). In post hoc analysis, the NH group had the highest rate of rapid naming, followed by 12–18 CI and 19–24 CI (p < 0.05) (Fig. 2, Table 3).

**Fig. 3:**
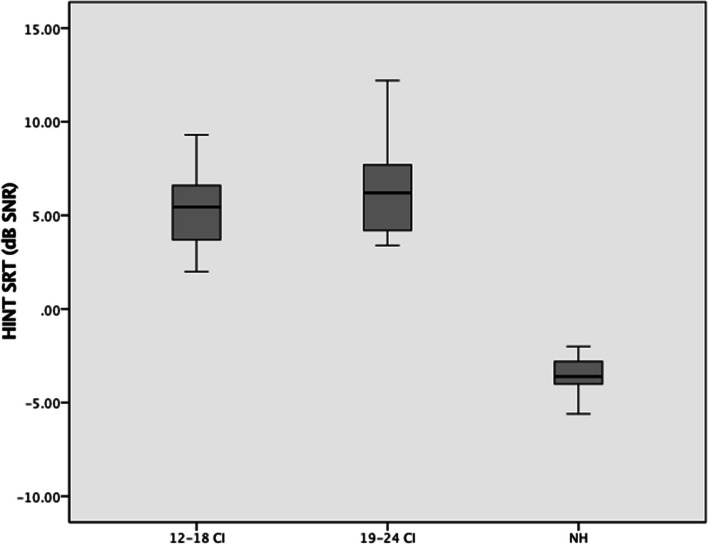
Comparison of HINT scores according to implantation period

In the HINT test, the mean SNR scores for the NH, 12–18 CI, and 19–24 CI groups were + 5.33 ± 2.08 (min: + 2.0, max: + 9.3), + 6.50 ± 2.52 (min: + 3.4, max: 12.2), and – 3.51 ± 0.91 (min: – 5.6, max: – 2.0). SNR scores differed significantly between groups. Post hoc analysis showed that the 12–18 CI and 19–24 CI groups had similar SNRs (*p* > 0.017), but the NH group had a significantly lower SNR (*p* < 0.017) (Fig. 3, Table 3).

### Comparison by unilateral/bilateral CIs

**Fig. 4:**
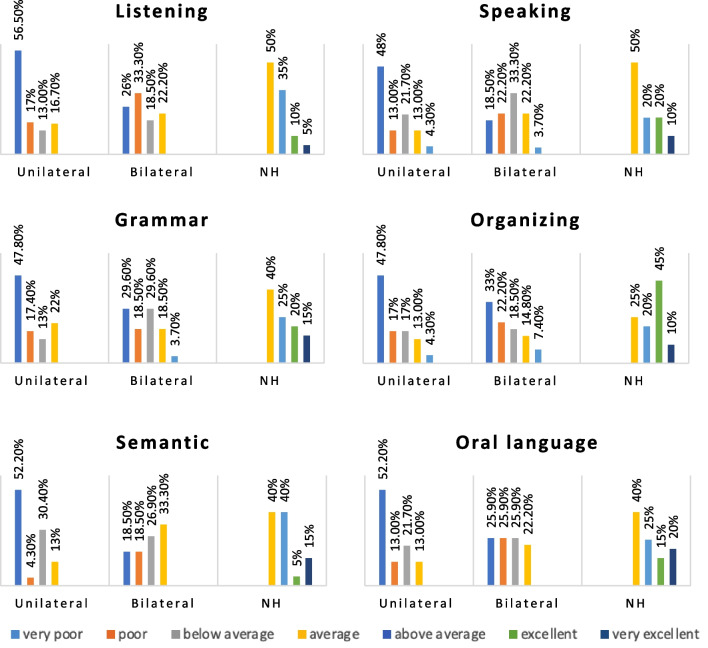
TOLD-P:4 category indexes according to unilateral/bilateral CI use

The UnCIs, BiCIs, and NH groups showed significant differences in the TOLD-P:4 indexes (listening, organizing, speaking, grammatical, semantic, and oral language) (*p* < 0.001). The post hoc analyses showed that the UnCI and BiCIs users' indexes categories were not significantly different (*p* > 0.017), but the NH group's categories were significantly higher (*p* < 0.017) (Fig. 4, Table 2).

**Fig. 5:**
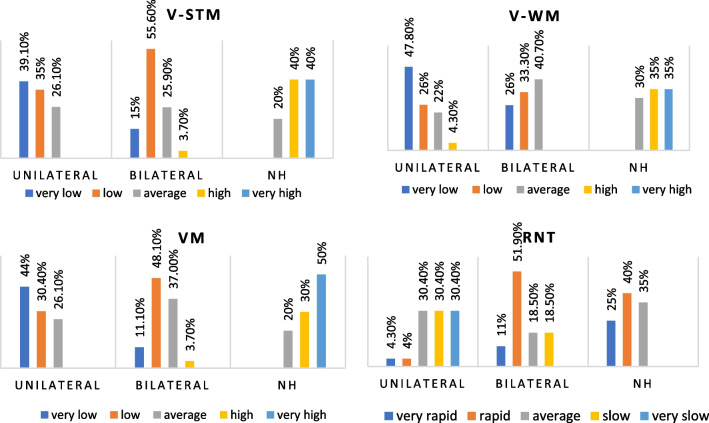
Comparison of VMS and RNT levels according to unilateral/bilateral CI use

In the WMS assessment, the groups' V-STM, V-WM, and VM levels were significantly different (*p* < 0.05). The post hoc analyses showed that the UnCIs and BiCIs users' STM, WM, and VM levels were not statistically different (*p* > 0.017), but the NH group's levels were significantly higher (*p* < 0017) (Fig. 5, Table 3).

The RNT showed that the groups' rapid naming levels were statistically different (*p* < 0.05). In the post hoc analysis, there was no statistically significant difference between UnCIs and BiCIs users (*p* > 0.017), while the NH group had a greater level of rapid naming than both groups (*p* < 0.017) (Fig. 5 , Table 3).

**Fig. 6:**
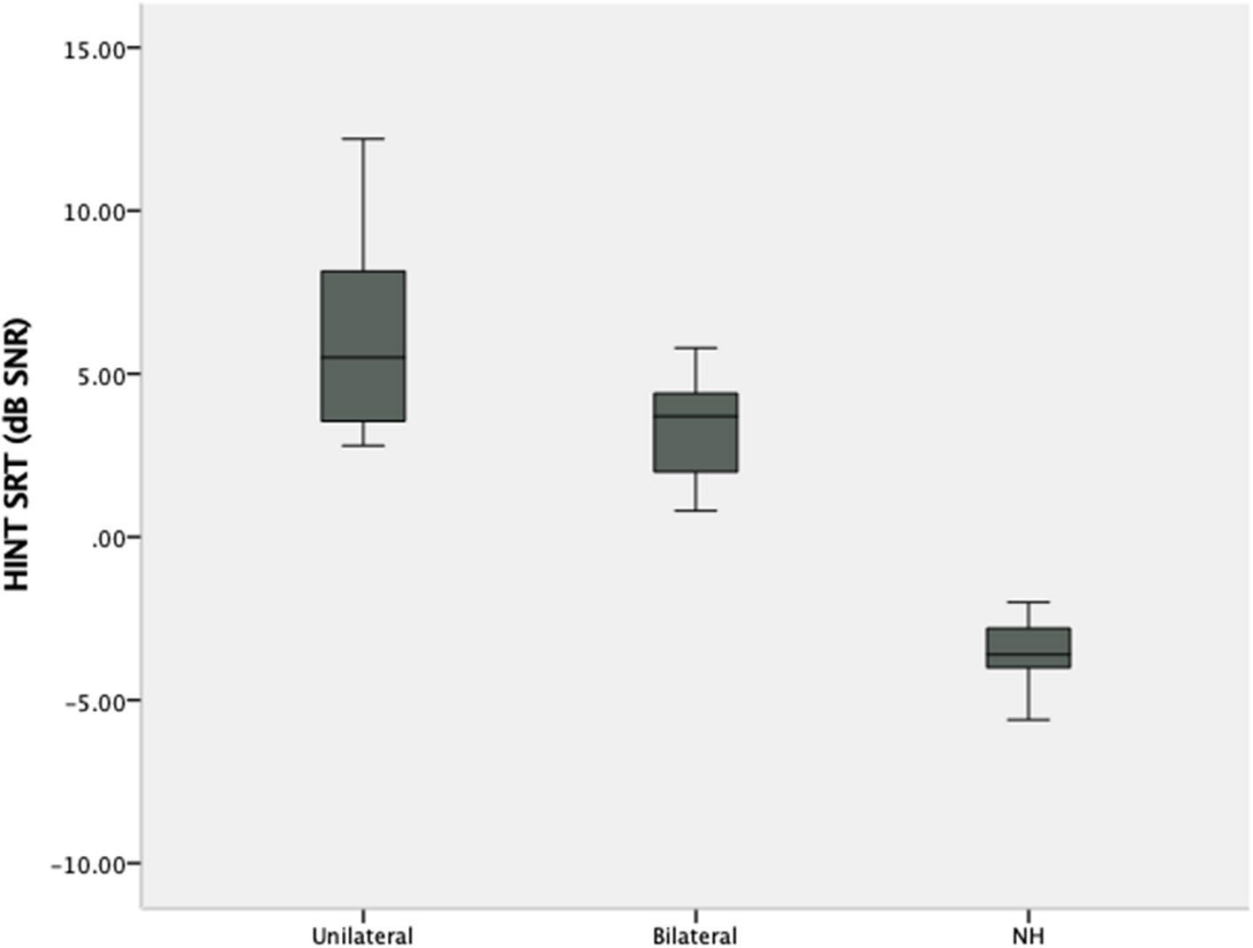
Comparison of HINT scores according to unilateral/bilateral CI use

In the HINT test, the mean SNR scores for the NH, UnCIs, and BiCIs groups were – 3.51 ± 0.91 (min: – 5.6, max: – 2.0), 6.03 ± 2.99 (min: 2.8, max: 12.2), and 3.35 ± 1.45 (min: 0.80, max: 5.80), respectively. Scores were statistically different between groups. The post hoc analyses revealed that the UnCIs users had the highest SNR, followed by BiCIs users, and then the NH group (Fig. 6, Table 3).

## Discussion

This study was conducted to compare the language, cognitive, and SiN perception abilities of CIs users and their NH peers according on implantation period and unilateral/bilateral implant use.

The primary benefit of CIs for children with prelingual deafness is the acquisition of the oral language. It also has a positive effect on other areas of development. However, cochlear implantation has mixed outcomes. Due to effects of HL on the developing auditory system and neuroplasticity, CI outcomes are strongly influenced by implantation age [22]. The FDA approved CIs for children as young as 12 months in 2000 and device-specific approval for 9-month-olds in 2020 [22, 33]. Thus, early implantation in the critical language development period reduces the consequences of auditory deprivation. The literature shows that children with CIs before 24 months of age are more likely to achieve age-appropriate auditory and language abilities [8, 18, 22]. In our country, cochlear implantation is approved for children from 12 months of age. We evaluated children who received CIs between 12 and 24 months. When we divided them into two groups based on their implantation period (12–18/19–24 months), we found no significant difference in their language skills, and they performed poorer than their NH peers. Robbins et al. [34] used a questionnaire to compare the auditory skills of children with CIs based on their implantation period (12–18/19–23/24–36 months) to their NH peers. In their study, parents of children with CIs were given the auditory questionnaire before, 3, 6, and 12 months after implantation. Auditory skills were not significantly different between 12 and 18 CIs and 19 and 24 CIs. However, they found that the youngest implanted children developed normal auditory skills sooner than children with CIs between 19 and 23 months. They also found that 19–23 months olds with CIs achieved auditory milestones earlier than 24–36 months old. At first glance, the findings of the current study suggests that there is no advantage to performing implantation in a child at 12–18 months of age, opposed to performing implantation at 19–24 months of age. However, this study is not longitudinal, we do not know language development rates. Nevertheless, the findings suggest that children with CHL who receive CIs between 12 and 18 months of age will not catch up to their NH peers in language skills. Because, by this time, NH children are familiar with their native language, have a receptive language foundation, and are starting to produce their first spoken words [35].

Children who receive CIs in early childhood represent a group of children who gain partial access to auditory speech information during the critical developmental period. The literature has debated whether early sensory input affects cognitive ability, particularly WM. Kronenberger et al. [36] examined spoken language and cognitive function in children with CIs and with NH. The majority of CI users were implanted between 1 and 2 years of age, and the others were implanted at 3 years or younger. They found that children with CIs scored more poorly than NH peers. Davidson et al. [37] examined the V-WM and vocabulary in CI and NH children. The children with CIs were implanted at 30 months of age or earlier. It was revealed that differences between the groups were more apparent for V-WM compared to visuo-spatial WM. In this study, children with CIs between 12 and 18 months and 19 and 24 months performed significantly worse on V-STM and V-WM tasks than their NH peers. The 12–18 CI group's V-WM level was higher than the 19–24 CI group's, but their V-STM levels were similar. Davidson et al. (2019) indicated that hearing deprivation during early development may impair VM storage and processing in children with CHL [37]. In addition, AuBuchon et al. [38] suggested that children with CIs may be less efficient at using phonological and linguistic strategies to maintain and process verbal information. These suggestions clarify our research's findings. Our findings demonstrated that children diagnosed with CHL and implanted between 12 and 18 months performed better in V-WM skills than their peers implanted between 19 and 24 months but poorer in total VM skills than their NH peers.

In this study, we also evaluated participants' rapid naming skill, as it is one of the most reliable method to assess long-term memory (LTM) access to lexical and phonological information. Because phonological processing is the foundation of language skills and LTM retrieves phonological representations of auditory stimuli in V-STM and V-WM [39]. Wechsler-Kashi et al. [40] suggested that LTM storage and retrieval could impede CI children's linguistic development. Because the store contains semantic, grammatical, motor, orthographic, and phonological information. Thus, we suggested that the age of implantation may affect rapid naming in children with CHL. According to our assumption, children who received their CIs between 12 and 18 months showed better rapid naming skills than those between 19 and 24 months. NH children also displayed better rapid naming than both groups. Unlike this study, Weschler-Kashi et al. [40] found no significant difference between CI children and their NH peers. It was noted that their study included children with varying HL onset ages. Our study included children with CHL. In their study, each picture was presented on a computer screen, and the child had 4 s to say the word. In difference, in the current study, all five items were presented in a repetitive and mixed order, and the child had 2 s to produce the word. Thus, even though prior research assessed the same ability, the approach and sample could explain such variations. Nicastri et al. [1] noted that despite the CI use, the phonological loop was frequently compromised in the SPHL. However, in the RNT, the high familiarity with the symbols may have compensated for the less effective functioning of the phonological loop. Nevertheless, this study revealed that children who had early CIs exhibited poor performance compared to their NH peers on the RNT and that the rapid naming skill is affected by the implantation age.

This study showed that children with CIs suffered language and cognitive impairments compared to their NH peers. Nicastri et al. [1] clarified in their comprehensive review that the limitations of V-STM might be related with the ongoing degradation of the representation of auditory input provided by the CI. Perceptual impairments in children with CIs may therefore impair the automatic retrieval of auditory information from the phonological storage area. As a result, it may be difficult to perform the storage, retrieval, and processing of partially encoded information appropriately. Additionally, limited speech perception may also make children with CIs more sensitive to irrevelant sound as well as less able to use indexical cue to help coding and storage of verbal material. Caldwell et al. [23] reported that children with CIs were worse at recognizing SiN than children with NH and HAs due to language and cognitive deficits. The Ease Language Understanding model explain that more difficult listening conditions require more robust linguistic knowledge, and if phonological or lexical representations are less detailed or unstable in children with HL, the degraded auditory input may not be sufficient for activating the correct lexical items [24]. In consistent with these informations, we found that children with CIs had a higher SNR to recognize SiN than children with NH. In addition, there was no significant difference between the SNRs of children implanted between the ages of 12–18 months and those implanted between 19 and 24 months. In considering the fact that the SiN perception is related to cognitive and language skills to predict the degraded stimulus, this findings were consistent with the previous findings of the study.

A growing number of deaf children receive BiCIs, either sequentially or simultaneously. Bilateral implantation improves spatial hearing, sound localization, and speech-to-noise separation for CI users in complex auditory situations [7, 21]. Thus, bilateral stimulation helps to reduce listening effort and facilitated incidental learning [7]. Wie et al. [7] stated that these benefits of BiCIs are supported by studies showing that children with bilateral CIs have better language outcomes than those with unilateral CIs. For example, Boons et al. [8] found that children with BiCIs achieved significantly higher receptive and expressive language scores than children with UnCIs, even though both groups received their CIs by the age of 2 years. Similarly to the current study, Yıldırım et al. [26] used to TOLD-P:4 to compare language skills in children with BiCIs and those with UnCIs. They found that children with BiCIs performed better in all language-based skills than children with UnCıs. Unlike these studies, we found no difference in language skills between children with BiCıs and UnCIs. NH children also were included and they showed significantly superior language skills than those with BiCıs and UnCIs. We assumed that the difference between the findings of our study and those of other studies was attributable to participant characteristics. For instance, in the study of Boons et al. (8), there were 25 UnCIs users and 25 BiCIs users. Eight children in the bilateral group received their implants simultaneously, while others received their implants sequentially. In the study of Yıldırım et al. [26], the interval time between surgeries in bilateral users was 4.77 ± 3.89 months. In the current study, children with BiCIs received their implants sequentially and the interval time between surgeries was 2,08 ± 0,86 years. Easwar et al. [41] stated that early simultaneous bilateral implantation promotes normal-like symmetry in auditory pathways. In addition, it is now widely accepted that in children with bilateral SPHL unilaterally implanted, the lack of an early implantation of the second ear would lead to the the hearing deprivation of one ear, due to a reorganization of the central auditory areas [42]. In this regard, the inter-implant delay seems to be a major factor of these findings, since early simultaneous bilateral cochlear implantation is the best option to promote oral language development for infants with SPHL [7, 42].

Understanding the mechanisms of speech encoding and processing is essential to understanding how BiCIs affect speech and language development in children with SPHL. Lee et al. [25] hypothesized that BiCIs may allow children with SPHL to allocate fewer cognitive resources to the encoding of phonological units and improve their speech perception and phonological representations. Their research confirmed this, showing that children with BiCIs outperformed than children with UnCIs on phonological awarenes, memory, and rapid naming tasks [25]. Despite their findings, we found that UnCIs children developed similar V-WM, V-STM, VM, and rapid naming skills to BiCIs children. NH children also had better V-WM, V-STM, VM, and rapid naming skills than children with BiCIs and UnCIs. However, Lee et al. [25] noted that their study's findings should be regarded with caution, because children with BiCIs wore their first HAs and received CIs earlier than children with UnCIs. They suggested that timing differences explain the difference between the groups. This study found no statistically significant difference between UnCIs and BiCIs children's first HA and CI ages. This strengthened our study's findings. Furthermore, BiCIs users received their CIs sequentially, which may explain the lack of difference between bilateral and unilateral users and their lower performance compared to NH peers.

In this study, there was no significant difference between bilateral and unilateral users' language, V-STM, V-WM, VM, and rapid naming abilities; nevertheless, there was a significant difference in their SiN perception. As we would expect, children with UnCIs require a higher SNR to perceive the SiN. In consistent with our findings, research has shown that bilateral cochlear implantation is superior to unilateral cochlear implantation for understanding SiN [43, 44]. Because, SiN perception is improved by having access to information from both ears, an effect described as the binaural benefit, which includes head shadow, summation, and squelch effects [43]. Although children with BiCIs develop the primary benefits of bilateral hearing, we found that children with BiCIs performed significantly more poorly than children with NH in recognizing SiN. Caldwell et al. [23] included children with NH, HA users, and simultaneous or sequential BiCIs users in their study and found that children with CIs performed poorly in speech recognition in noise and quiet compared to children with NH and HAs users. These results were explained by the fact that CIs only provide a sparse signal representation, lacking many of acoustic properties, especially spectral ones. In addition to these technological limitations reported by Caldwell et al. [23], the results in our study can also be partially explained by biological limitations; that is, binaural skills may be compromised due to expanded unilateral deafness caused by sequential bilateral cochlear implantation.

In this study, we included a homogenus CIs group in terms of auditory experience (age at diagnosis, age at HA fitting, and age at first CI), receiving regular implant mapping and auditory rehabilitation our center, and receiving special education regularly after first CI. It was believed that the inclusion of the homoegenous group and comparison of their measurement results with those of their NH peers contributed to the strength of the study. The fact that the participants received education from different professionals in different special education centers in addition to our center and that the content of the rehabilitation programs were unknown was considered as the study's major limitation. Another limitation of this study is that simultaneous BiCIs users were not included due to their very small number. In future studies, it is recommended to include early simultaneous BiCIs users as a separate group and compare their language, cognitive and SiN perception abilities with early sequential BİCIs users and UnCI users.

In conclusion, early diagnosis and early intervention are crucial for the different developmental abilities of children with CHL. However, this study showed that in children with CHL, cochlear implantation between 12 and 18 months or sequential bilateral implantation is not sufficient for these children to perform like their NH peers in language, cognitive, and SiN perception abilities. In addition, the present study highlights that a more complete understanding of the strengths and limitations of children with CI in different developmental areas is crucial for planning therapy. Therefore, intervention approaches should focus not only on increasing language skills, but also on cognitive skills. That is, it seems important to tailor intervention for each child by matching therapy details of their linguistic and cognitive knowledge.

## Data Availability

Not applicable.
